# Monoterpenes in Vascular Function: A Review of Bioactivity and Mechanisms of Action

**DOI:** 10.3390/ijms26189243

**Published:** 2025-09-22

**Authors:** Tays Gonçalves, Arthur Almeida, Larisse Pontes, Julio Oliveira, Mathania Feitosa, Javanyr Júnior, Robson Veras, Isac Medeiros

**Affiliations:** Department of Pharmaceutical Sciences, Federal University of Paraiba, João Pessoa 58059-900, PB, Brazil; taysamanda@ltf.ufpb.br (T.G.); arthur-jp@hotmail.com (A.A.); larisse.virgolino@ltf.ufpb.br (L.P.); juliopinheiro@ltf.ufpb.br (J.O.); mathania_rezende@hotmail.com (M.F.); javanyrjunior@ltf.ufpb.br (J.J.); robsonveras@ccs.ufpb.br (R.V.)

**Keywords:** protective effect, monoterpenes, vascular health, vascular disease, vascular protection, cardiovascular disease

## Abstract

Cardiovascular diseases are the primary cause of morbidity and mortality worldwide. The function and structure of blood vessels play a crucial role in the development and aggravation of these diseases. Natural products, such as aromatic plants, present a wide variety of terpenes content. Monoterpenes, a selected group of terpenes, have two building blocks of five-carbon isoprene (C_5_H_8_) unit. Moreover, different monoterpenes have shown pharmacological activity in the cardiovascular system, particularly in vascular function, which is mediated, at least in part, by modulating the nitric oxide pathway, oxidative stress, inflammation, and calcium signaling. Therefore, this review addresses the role of monoterpenes as pharmacological tools in the vascular system, providing mechanisms of action and their biological effects.

## 1. Introduction

Aromatic plants have been widely used for their medicinal properties over the centuries. Essential oils—formed mainly by terpene derivatives representing approximately 90%—are abundant volatile compounds in aromatic plants [1]. Monoterpenes represent formula C_10_H_16_, which represents an important class of secondary metabolites known for their diverse pharmacological activities. These activities include antioxidant, anticancer, anti-inflammatory, anti-senescence, neuroprotective, and antidiabetic effects [2,3,4]. In addition, monoterpenes improve cardiovascular function by targeting vascular cells such as endothelium and smooth muscle cells [1,5,6].

It is worth noting that monoterpenes can be functionalized by the addition of functional groups (e.g., ether, ester, and epoxide) [7]. They can also be used as precursors for the synthesis of isoprenoid natural products, as demonstrated for (−)-sclareol [8]. Furthermore, several advanced delivery systems have been developed to increase stability, bioavailability, and controlled release, allowing for their safer clinical use [9].

Although the clinical development of many monoterpenes remains limited, the preclinical biochemical and pharmacological properties of many of these secondary metabolites have been extensively investigated, as highlighted in this review.

In this review, we draft an overview of the most widely studied monoterpenes, including their sources, synthesis, biological effects, and advances in elucidating the molecular and physiological pathways to prevent/delay vascular pathologies.

## 2. Monoterpenes Chemistry: An Overview

Terpene hydrocarbons have a molecular formula of (C_5_H_8_)n; n dictates the number of units involved. The building block is a five-carbon isoprene (C_5_H_8_) unit. Therefore, terpene hydrocarbons are classified according to the number of isoprene units (C_5_): hemiterpenes (C_5_), monoterpenes (C_10_), sesquiterpenes (C_15_), diterpenes (C_20_), sesterterpenes (C_25_), triterpenes (C_30_), and carotenoids (C_40_).

**Figure 1:**
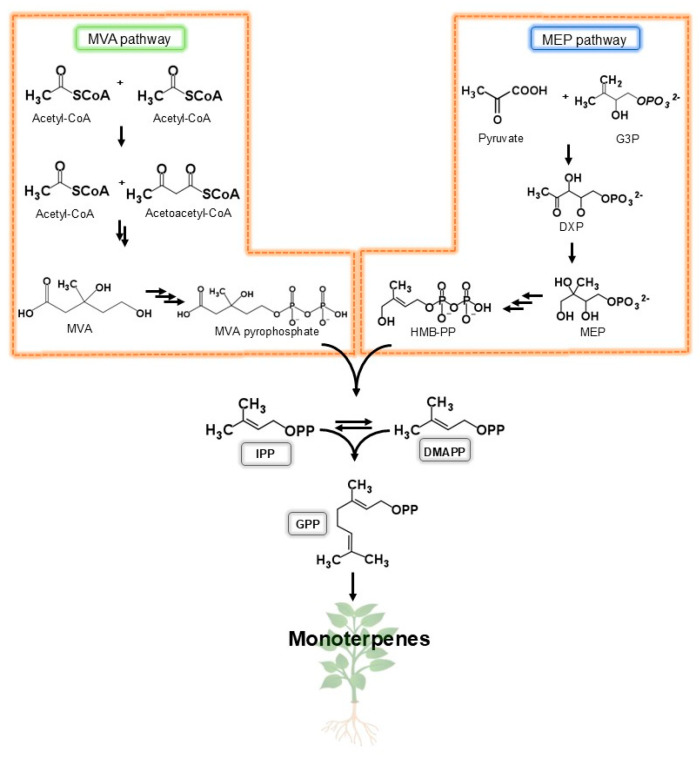
The chemical structures and biosynthesis pathways of monoterpenes. Legend: Glyceraldehyde-3-phosphate (G3P); 1-deoxy-d-xylulose 5-phosphate (DXP); methylerythitol 4-phosphate (MEP); 4-hydroxy-3-methyl-butenyl 1-diphosphate (HMB-PP); mevalonate (MVA); isopentenyl pyrophosphate (IPP); dimethylallyl pyrophosphate (DMAPP); and geranyl pyrophosphate (GPP).

Monoterpene biosynthesis depends on two biochemically active isoprene units: isopentenyl diphosphate (IPP) and its dimethylallyl diphosphate isomer (DMAPP). The IPP and DMAPP are synthesized through the mevalonate pathway (MVA) or the 4-phosphate methylerythritol pathway (MEP), also known as 1-deoxy-d-xylulose 5-phosphate (DXP) [9]. For the formation of mevalonic acid, the condensation of three molecules of acetyl-CoA occurs, since the initiators of the MEP are pyruvate and glyceraldehyde 3-phosphate [10] (Figure 1). Subsequently, the enzyme geranyl diphosphate (GPP) synthase acts by condensing the IPP and DMAPP, which produce GPP, a common precursor for all monoterpenes [11]. Different enzymes target GPP to promote isomerization, addition, and elimination reactions, enhancing monoterpenes variety [12].

**Figure 2:**
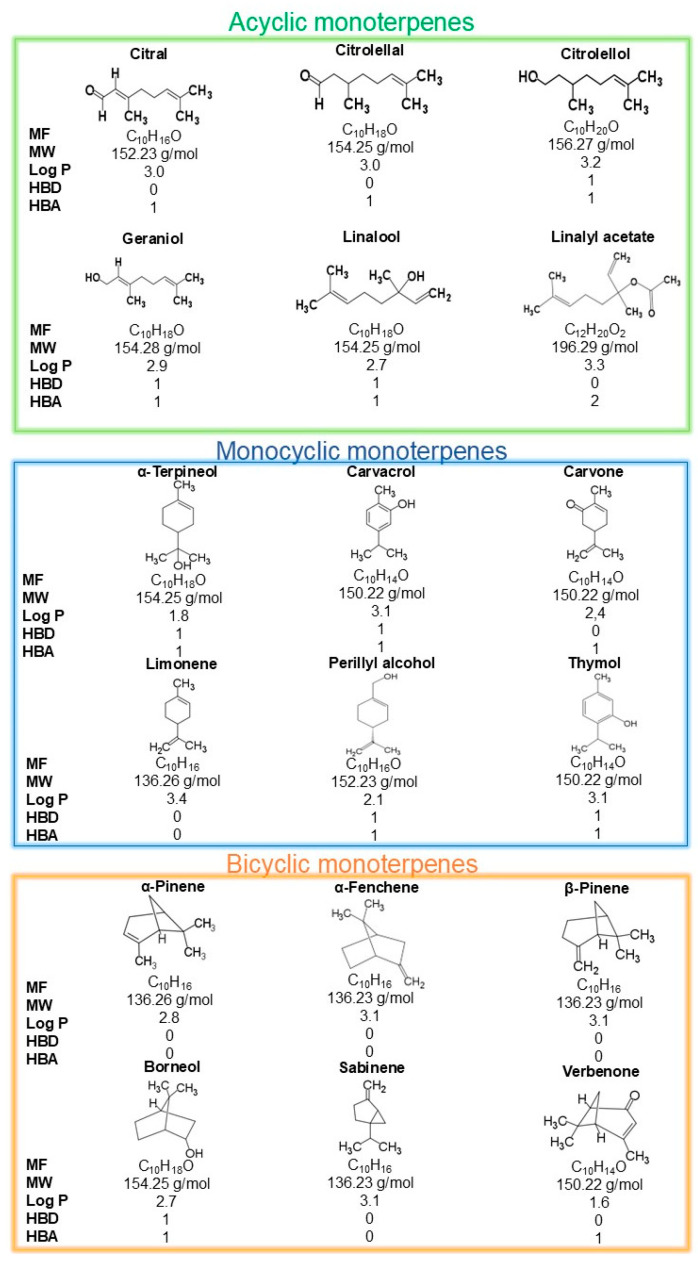
The classification and physical properties of monoterpenes. Legend: Molecular Formula (MF); Molecular Weight (MW); Logarithm of the partition coeficiente (Log P); Hydrogen Bond Donor Count (HBD); and Hydrogen Bond Acceptor Count (HBA).

Monoterpenes are generally classified according to their carbon skeleton, types of cyclization, and oxidation state [4]. The structural variations in monoterpenes form subclasses, which can be acyclic or cyclic. Acyclic compounds have a linear structure like a tail-head arrangement of isoprene units. In contrast, cyclic compounds are divided into monocyclic (one cycle) or bicyclic (two cycles) (Figure 2). Moreover, cyclization occurs through monoterpene cyclase enzyme activities in multistep reactions, producing several compounds [13].

In addition, based on the oxidation state and functional group diversity, monoterpenes can be classified into two subgroups: hydrocarbons and oxygenated monoterpenes. Hydrocarbon monoterpenes do not have oxygen in their structure and have an alkene hydrocarbon moiety. However, oxygenated monoterpenes may differ according to their functional group, such as hydroxyl groups (alcohols and phenols), carbonyl-containing groups (aldehydes, ketones, carboxylic acids, carboxylic esters, and lactones), and groups with oxygen bridges (ethers, peroxides, and furans) [4,14].

## 3. Role of Monoterpenes on the Vascular Function

Blood vessels are heterogeneous structures of three layers, including the intima, which comprises a single layer of endothelial cells—the media, predominantly of smooth muscle cells, and elastic fibers. Finally, the adventitia with fibroblasts, collagen fibers, and perivascular nerves [15]. Each layer exhibits specific histological, biochemical, and functional characteristics, and each contributes uniquely to maintaining vascular integrity.

Under physiological conditions, the endothelium modulates the vasodilatory, thrombolytic, immune, and vasoprotective functions by synthesizing and releasing specific substances that act locally and systemically [16].

The endothelium regulation of vascular tone evolves relaxation and contraction mechanisms in the underlying vascular smooth muscle. Thereby, endothelial cells secrete both vasodilator [nitric oxide (NO), prostacyclin, endothelium-derived hyperpolarizing factor] and vasoconstrictor substances (angiotensin II, endothelin-1, thromboxane A_2_) depending on stimulus [17].

**Figure 3:**
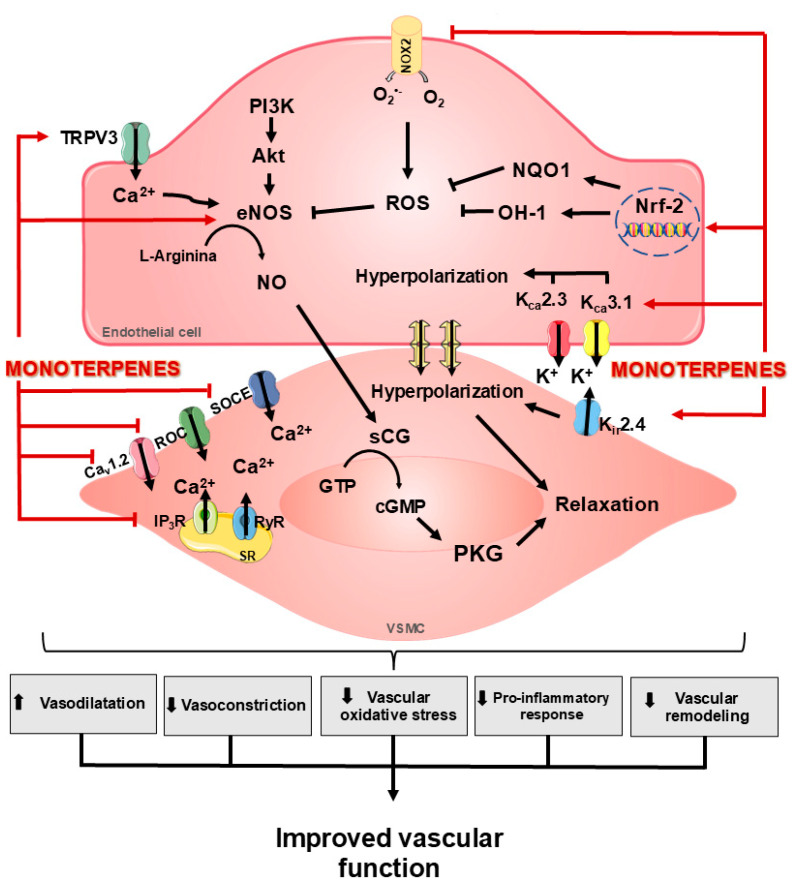
Protective effect of monoterpenes on the blood vessel. Monoterpenes promote vasodilation and vascular protection through multiple mechanisms: they increase the expression and activation of eNOS via Akt/PKB phosphorylation. Monoterpenes also act on TRPV3, increasing Ca^2+^, which activates eNOS in endothelial cells. NO production activates GCs, increasing cGMP formation and, consequently, leading to VSMC relaxation. Furthermore, monoterpenes reduce ROS formation, which culminates in increased NO bioavailability. Monoterpenes also activate K_Ca_2.3 and KCa3.1 channels, inducing hyperpolarization in endothelial cells. Monoterpene-mediated endothelial hyperpolarization propagates to VSMC through gap junctions, causing relaxation. Furthermore, monoterpenes modulate channels such as Cav1.2, ROC, and SOCE in VSMC, reducing Ca^2+^ entry and inducing relaxation. Legend: Reactive oxygen species (ROS); Heme oxygenase-1 (HO-1); Quinone oxidoreductase-1 (NQO1); Phosphoinositide-3-kinase (PI3K); Protein kinase B (Akt or PKB); Endothelial nitric oxide synthase (eNOS); Nitric oxide (NO); Soluble guanylyl cyclase (sGC); Guanosine triphosphate (GTP); Cyclic guanosine monophosphate (cGMP); Receptor-operated calcium channels (ROC); Store-operated calcium entry (SOCE); Inositol 1,4,5-trisphosphate receptor (IP3R); Voltage-gated L-type calcium channels (Cav1.2); ATP-sensitive potassium channels (Kir2.4); potassium channels activated by small (K_Ca_2.3—SK_Ca_) and intermediate (K_Ca_3.1—IK_Ca_); Sarcoplasmic reticulum (SR); and Vascular smooth muscle cells (VSMC).

The balance of such mechanisms is essential for ensuring proper maintenance of vascular tone and function. In contrast, pathological conditions lead to vasoactive factor-mediated response dysfunction. Thus, the vasoconstrictor, prothrombotic, and pro-inflammatory state is favored, leading to vascular impairment [18]. Furthermore, different studies have demonstrated the vasoprotective action of monoterpenes, modulating vasodilator, anti-inflammatory, antioxidant, and vascular remodeling responses [2,3,4]. Therefore, the section below will describe the primary mechanisms by which monoterpenes induce these effects (Figure 3).

### 3.1. Effects of Monoterpenes on NO Signaling and Oxidative Stress

NO, a short-lived gaseous molecule, is one of the primary vasodilators generated by the endothelium [19]. In physiological conditions, NO is produced in the presence of oxygen and l-arginine via endothelial nitric oxide synthase (eNOS), using tetrahydrobiopterin (BH_4_) as a cofactor [20]. In addition, eNOS activation can occur by acetylcholine and bradykinin stimulation [21] and through eNOS phosphorylation on Ser1177, which is mediated by the phosphatidylinositol 3-kinase (PI3K)/Akt signaling pathway [16]. Thus, NO diffuses into the underlying VSMC, activating soluble guanylyl cyclase (sCG) and producing cyclic guanosine monophosphate (cGMP), which results in vasodilation [22].

Monoterpenes mediate their effects, at least in part, by the modulation of NO synthesis, which involves eNOS activity. Recent findings demonstrated by experiments in the presence of L-NAME, an eNOS inhibitor, that monoterpene effects were abolished [23,24]. Moreover, monoterpenes-induced treatment increased eNOS expression, resulting in NO release [25,26]. Chen and collaborators showed that monoterpenes act by increasing Akt phosphorylation, resulting in eNOS phosphorylation and activation [27]. Furthermore, monoterpenes also act downstream in the NO signaling pathway. Experiments in the presence of 1H-[1,2,4]oxadiazole [4,3-a]quinoxalin-1-one (ODQ), an sCG inhibitor, the vasodilator response induced by monoterpenes was significantly reduced, demonstrating the participation of the NO–sCG–cGMP pathway in monoterpenes’ effects [28,29].

Moreover, ROS can alter vascular function through changes in vascular tone, mainly by reducing the NO bioavailability, favoring the contractile state [18,20,30]. However, several monoterpenes have antioxidant capacity, and experiments evaluating the isometric tensions of blood vessels to prooxidant agents such as mercury, arsenic, and lead revealed that monoterpenes were able to improve the relaxing response impaired by these agents. Thus, the antioxidant capacity of monoterpenes resulted in ROS depletion and increased NO bioavailability [31,32].

### 3.2. Effects of Monoterpenes on K^+^ and EDHF Channels

Monoterpenes modulate K^+^ channels by the NO–sCG–cGMP pathway or through direct modulation of different channels. Evidence suggests that the presence of K^+^ channel inhibitors attenuates monoterpene relaxation. Monoterpenes act on different channels, mainly involving voltage-operated K^+^ channels (K_v_) [33], ATP-sensitive potassium channels (Kir2.4) [23], potassium channels activated by small (K_Ca_2.3—SK_Ca_) and intermediate (K_Ca_3.1—IK_Ca_) calcium conductance [34].

Moreover, recent findings suggest that K_Ca_2.3 and K_Ca_3.1, present in the endothelium, produce a hyperpolarizing response that spreads to adjacent VSMC through endothelial gap junctions [35,36]. These channels are classified into endothelium-derived hyperpolarizing factors (EDHF) [22,37]. The hyperpolarizing effect generated by monoterpenes is blocked by TRAM34, a K_Ca_3.1 inhibitor, and apamin, a K_Ca_2.3 inhibitor [34]. Thus, activation of EDHF-mediated relaxation is an essential regulatory pathway mediated by monoterpenes.

### 3.3. Effects of Monoterpenes on TRP Channels and Ca^2+^ Signaling

Monoterpene activates the vanilloid receptor TRPV3 [38], which is highly permeable to Ca^2+^ [39]. Monoterpenes activate the vanilloid receptor TRPV3 [38], which is highly permeable to Ca^2+^ [39]. In endothelial cells, monoterpene-mediated TRPV3 activation increases intracellular Ca^2+^ concentrations. This Ca^2+^ influx culminates in the activation of calcium-sensitive K^+^ channels, such as K_Ca_2.3/K_Ca_3, leading to hyperpolarization and subsequent vascular relaxation [40,41]. Corroborating these results, the vasorelaxant effect induced by monoterpenes was inhibited by endothelium removal and abolished by pharmacological blockade of K_Ca_2.3 and K_Ca_3.1 channels. These results suggest that the Ca^2+^ influx required for activation of these channels depends on the response evoked by TRPV3 [40].

Furthermore, TRPs regulate vascular tone by mediating responses by modulating critical pathways in SMC [42]. Interestingly, the participation of TRP in vessels with absent endothelium highlights that monoterpenes can modulate the activity of TRPM7, TRPC1 [43], and TRPM8 [44].

Dantas and collaborators demonstrated that in the presence of magnesium at a concentration of 2.5 mM, which inhibits TRPM7 activity, the TRPM7 channel participates in the relaxant response [43]. Similarly, experiments performed in the presence of BCTC (4-(3-Chloro-2-pyridinyl)-N-[4-(1,1-dimethylethyl)phenyl]-1-piperazinecarboxamide), a potent TRPM8 inhibitor, the terpene-induced relaxing response was drastically reduced [44]. Therefore, these data suggest that monoterpenes may act by blocking TRPM7 and TRPM8 to induce vasorelaxation.

A key target in regulating vascular tone is calcium ion signaling in VSMC [45]. Calcium concentration in these cells is regulated via voltage-gated L-type calcium channels (Ca_v_1.2), receptor-operated calcium channels (ROC), or released calcium from intracellular stores. In addition, several monoterpenes can alter calcium influx into VSMC through direct or indirect inhibition of these channels.

In addition, TRPC1 is a molecular component of Store-operated calcium entry (SOCE), a mechanism for Ca^2+^ entry across the plasma membrane when intracellular Ca^2+^ stores in the endoplasmic reticulum are depleted [46]. TRPC1 participation in this effect was evaluated using intracellular deposits of Ca^2+^ depletion, maintained with a depolarizing solution and with the addition of nifedipine, a Ca_v_1.2 inhibitor, and cyclopiazonic acid, a SERCA inhibitor, observing a reduction in the contraction induced by CaCl_2_. Moreover, corroborating the participation of TRPC1, experiments were performed in the presence of TRPC1 channel blockers lanthanum (100 μM) and gadolinium (100 μM). Under these conditions, a potential to recover from the relaxing response was observed, confirming an interaction between TRPC1 channels and monoterpenes in VSCM [43].

Furthermore, in the presence of KCl, a reduction in contractile force and intracellular calcium concentration was observed [47]. Likewise, pre-incubation with terpenes reduced the fluorescence of Fluo-4 AM—a probe related to calcium concentrations—induced with CaCl_2_, similar to the nifedipine effect [48]. In addition, patch-clamp electrophysiological experiments demonstrated that monoterpenes reduce Ca_v_1.2-mediated calcium currents in VSMCs [34,43,49,50]. Thus, Ca_v_1.2 is an essential target for pharmacological studies of monoterpenes.

## 4. Beneficial Effects of Monoterpenes on Vasculature

Monoterpenes and their derivatives are promising molecules as new therapeutic agents, especially in cardiovascular diseases [3,6]. Numerous monoterpenes have shown activity in the cardiovascular system, particularly in vascular function, which is mediated, at least in part, by modulating the nitric oxide pathway, oxidative stress, inflammation, and calcium signaling. Therefore, describing the monoterpenes-inducing vasoprotective effect is appropriate. Additionally, the vascular effects of monoterpenes are briefly described in Table 1.

### 4.1. Geraniol

Geraniol is a monoterpene alcohol isolated from aromatic plants, including *Cinnamomum tenuifolium* and *Valeriana officinalis*. Geraniol presents a range of biochemical and pharmacological properties, such as anticancer [2], antioxidants, anti-inflammatory [77], and cardiovascular effects [78]. Its therapeutic relevance is particularly evident in the context of endothelial dysfunction characteristic of conditions such as diabetes, metabolic syndrome, and atherosclerosis, where it demonstrates multifaceted effects.

Vascular complications are a major cause of death associated with cardiovascular disease. In diabetes and metabolic syndrome, potentiation of the vasoconstrictor response and significant impairment of Ach-induced vasorelaxation are observed [79,80]. These effects are often associated with the formation of advanced glycation end products (AGEs), which reduce NO bioavailability [81,82].

Importantly, geraniol demonstrated the ability to reduce hypercontractility and improve ACh-mediated vasodilation in the aorta of diabetic rats or those with metabolic syndrome, indicating that it can improve vascular dysfunction. The mechanism of geraniol-mediated vasorelaxation was also evaluated in the aortas of diabetic rats and primarily involves the blockade of Cav1.2 and ROC calcium channels, highlighting a direct effect on vascular smooth muscle [51]. These findings corroborate the reduction in hypercontractility observed in the studied models.

Endothelial dysfunction is widely accepted as the first sign of atherosclerosis, a condition in which inflammation and oxidative stress play central roles in pathogenesis [83]. ROS impair NO bioavailability, playing a vital role in vascular damage. In a high-fat diet model (mimicking atherosclerosis in vivo), geraniol was able to restore ACh-induced endothelium-dependent vascular relaxation [68]. Furthermore, geraniol reduced oxidative stress induced by superoxide anions and reduced the expression of NOX2 [68].

Geraniol appears to exert effects by stimulating the synthesis of antioxidant factors. In HUVECs treated with OX-LDL, an in vitro model of atherosclerosis, geraniol activated the PI3K/AKT/NRF2 pathway, increasing HO-1 expression [52]. These results collectively reveal geraniol’s potential antioxidant role in improving endothelial dysfunction.

Inflammation is also a key determinant in atherosclerotic plaque initiation and progression. Under atherogenic stimuli, endothelial cells express inflammatory cytokines (e.g., tumor necrosis factor-alpha (TNF-α), interleukin (IL)-8) and adhesion molecules (e.g., intercellular adhesion molecule-1 (ICAM-1), vascular adhesion molecule-1 (VCAM-1), E-selectin, P-selectin). These attract lymphocytes and monocytes that bind to the endothelium and infiltrate the arterial wall [84]. 

In HUVECS treated with OX-LDL, geraniol inhibited the production of pro-inflammatory cytokines, including TNF-α, IL-6, and IL-1β, and suppressed the nuclear translocation and activity of NF-κB [52]. Similar results were observed by Wang et al. in the spinal cord injury (SCI) model, a model of inflammation. Under these conditions, geraniol reduced serum levels of TNF-α, IL-6, and IL-1β, as well as the expression of NF-κB, VCAM-1, and ICAM-1, confirming its anti-inflammatory properties [85].

Therefore, the findings indicate that geraniol’s endothelial protection primarily derives from its antioxidant and anti-inflammatory effects, holding particular clinical relevance for metabolic conditions associated with vascular dysfunction (diabetes, metabolic syndrome, atherosclerosis). However, the lack of human studies limits the extrapolation of these findings to therapeutic applications.

### 4.2. Carvacrol

Carvacrol is a phenolic monoterpenoid found in essential oils of thyme (*Thymus vulgaris*), oregano (*Origanum vulgare*), pepperwort (*Lepidium flavum*), and wild bergamot (*Bergamia Loise* var. *Citrus aurantium*) [86]. Carvacrol has been shown to have several benefits, including antioxidant [87], anti-inflammatory [88] properties. It has been highlighted for its multiple beneficial effects on the cardiovascular system [27,43,89], including vasoprotective actions with mechanisms of action already characterized in experimental models.

Carvacrol has been shown to exert significant structural and functional effects on vascular function in diabetes [69,90]. Macrovascular and microvascular complications are prevalent and severe among diabetic patients. These complications are associated with endothelial dysfunction, which is characterized by increased inflammation and oxidative stress, and decreased NO bioavailability, which consequently induces structural changes in blood vessels [83]. These changes are associated with the potentiation of hypercontractility and impaired relaxation.

In an in vivo model of streptozotocin-induced diabetes, Liu and colleagues observed that carvacrol was able to reverse the pathological vascular remodeling of diabetes and inhibited vascular hypercontractility in aortic arteries. Furthermore, carvacrol treatment stimulated the activation of the PI3K/Akt signaling pathway in diabetic animals, which consequently increased NO production [69].

The anti-inflammatory effects of carvacrol on diabetes were evaluated in C57BL/KsJ mice, a type 2 diabetes model with obesity, and in HUVECs exposed to high glucose concentrations. In this animal model of diabetes, carvacrol was also able to reverse the macrovascular complications associated with diabetes. These effects are associated with its potent anti-inflammatory action, which includes inhibition of the inflammatory transcription factor NF-κB pathway and consequent reduction in the production of pro-inflammatory cytokines such as TNF-α, IL-8, and IL-1β, both in vitro and in vivo studies [90].

Hemodynamically, carvacrol has well documented hypotensive and vasodilatory properties, mediated primarily by blockade of Cav1.2 channels and modulation of TRP channels [43]. However, other studies report divergent results, revealing that carvacrol’s vasodilatory response appears to involve potassium (Kv) channels, as its efficacy is significantly reduced by Kv channel blockers [33].

Carvacrol also demonstrates a remarkable ability to improve endothelial function by modulating oxidative stress. In models of lead toxicity, the compound was able to restore endothelium-dependent vasorelaxation, a mechanism related to the reduction in ROS and increased NO bioavailability [31].

Regarding the pathological endothelial dysfunction of arterial hypertension, carvacrol demonstrates additional therapeutic potential in the mobilization of endothelial progenitor cells (EPCs), promoting the reduction in oxidative stress and vascular repair. This effect is associated with improved EPC progenitor function, with reduced oxidative stress and cellular senescence [70].

In summary, carvacrol exhibits vascular protective effects by targeting key pathological mechanisms in diabetes and hypertension, including oxidative stress, inflammation, endothelial dysfunction, and vascular remodeling. These effects position the compound as an attractive candidate for clinical trials to consolidate its therapeutic potential.

### 4.3. Citronellal

Citronellal is a monoterpene extracted from the oils of *Corymbia citriodora*, *Cymbopogon nardus*, *Cymbopogon citratus*, and *Cymbopogon winterianus*, typically isolated as a racemic mixture of its R and S enantiomers [91]. Citronellal has been recognized for its pharmacological actions, which include anti-inflammatory [92], antioxidant [73], cardioprotective [93], and vasoprotective [71,72] effects, with significant benefits for endothelial dysfunction.

Experimental studies demonstrate that citronellal exerts marked antiatherosclerotic effects, reducing plaque size in animal models and significantly improving endothelial function. These effects were demonstrated in a high-fat diet model [71] and streptozotocin-induced diabetic rats [73,94]. In both models, citronellal improved oxidative stress and vascular inflammation. The primary mechanism for reducing atherosclerotic plaque formation involves inhibition of the Na^+^/H^+^ exchanger type 1 (NHE1) [71,73].

Recent studies have demonstrated an important role for the NHE1 exchanger in the development of atherosclerosis. This exchanger is hyperactivated in some pathophysiological conditions, leading to acidification of atherosclerotic lesions and increased endothelial cell apoptosis [95]. Thus, by inhibiting NHE1, citronellal prevents atherosclerotic lesion formation.

Complementing this primary action, citronellal acts synergistically through the modulation of TRPM2 [73]. TRPM2 is a calcium ion-permeable cation channel that is activated in response to oxidative stress, causing further endothelial dysfunction [96]. Furthermore, overexpression of TRPM2 can increase NHE1 expression. Interestingly, in HUVEC exposed to high glucose, citronellal was able to reverse the effects mediated by TRPM2 overexpression [73]. This dual action proves particularly effective in protecting endothelial function.

In the streptozotocin-induced diabetes model, citronellal demonstrates a third mechanism via sphingosine 1-phosphate (S1P1) receptor activation [72]. S1P1, highly expressed in endothelial cells, mediates physiological functions like cytoskeletal organization, migration, and vascular maturation [97]. Its signaling is impaired under high glucose. Qiu and colleagues showed citronellal increases S1P1 expression, which in turn increases eNOS expression and NO production while reducing oxidative stress. These effects collectively improve vasodilation in diabetic thoracic aortas [72].

Despite the very positive effects of citronellal on vascular function, significant challenges remain for the clinical translation of these findings. Issues such as the possible difference in activity between the R and S enantiomers, the determination of optimal therapeutic windows, and validation in human models of cardiovascular disease require further investigation.

### 4.4. Citronellol

Citronellol is found abundantly in plants of the genus *Cymbopogon* and *Citrus*. It has an amplitude of biological effects, such as anti-inflammatory and antioxidant [98,99]. Recent research demonstrates its action on the cardiovascular system, describing the molecular mechanisms responsible for its antidiabetic [100], hypotensive and vasorelaxant action [55].

Pharmacological studies demonstrate that citronellol exerts significant cardiovascular effects through several integrated mechanisms. Intravenous administration of citronellol induced a hypotensive response associated with reflex tachycardia [55]. Regarding vascular tone, citronellol exerts a concentration-dependent, endothelium-independent relaxing effect. The underlying mechanism involves Cav1.2 activation and calcium mobilization from intracellular stores [55,101].

The benefits of citronellol also extend to protection against ischemic damage. In a stroke model (intraluminal middle cerebral artery occlusion/reperfusion (MCAO/R)), citronellol treatment significantly increased antioxidant enzymes such as SOD, CAT, and glutathione. Furthermore, levels of pro-inflammatory cytokines such as TNF-α, IL-1, and IL-6 were reduced, while anti-inflammatory cytokines such as IL-4 and IL-10 increased [102].

Therefore, based on the studies conducted, citronellol reveals that its cardiovascular effects result from its direct action on vascular tone and its antioxidant and anti-inflammatory properties. However, further studies are needed to explore its role in vascular function and diseases associated with blood vessels.

### 4.5. Linalyl Acetate

Linalyl acetate is a monoterpene present in several species of aromatic plants, such as the citrus genus *Citrus bergamia risso* (bergamot), *Lavandula angustifolia* (lavender), and *Salvia sclarea* (clary sage) [26]. Linalyl acetate has emerged as a promising compound in cardiovascular protection due to its anti-inflammatory effects [103].

The vasorelaxant mechanism of linalyl acetate was evaluated in the carotid artery of white male rabbits. In arteries with functional endothelium, this monoterpene promoted a partial relaxing effect by stimulating the production of NO and activating sGC in the vascular smooth muscle. On the other hand, linalyl acetate induces activation of myosin light chain phosphatase (MLCP) [56], reducing the contractility of the vascular smooth muscle. This action of Linalyl acetate differentiates it from many vasodilators, giving a therapeutic advantage in some pathological conditions.

The beneficial effects of Linalyl acetate were observed in a hypertension model using the immobilization model combined with nicotine administration, an ischemic-hypertensive injury model. This model was used to mimic chronic stress associated with the use of nicotine, which was able to induce a significant increase in blood pressure and vascular dysfunction.

In this model, Linalyl acetate was able to decrease blood pressure and demonstrated the ability to prevent endothelial damage, partially restoring eNOS protein levels and, consequently, increasing the production of NO [26]. This study corroborates the previously reported results that demonstrated that Linalyl acetate in the nicotine model increases the production of NO [104].

Linalyl acetate was also evaluated in the hypertension model associated with chronic obstructive pulmonary disease (COPD) (induced simultaneously by intranasal administration of porcine pancreatic elastase (PPE) and lipopolysaccharide (LPS), along with chronic exposure to nicotine stress and immobilization), characterized by significant blood pressure and increased serum LDH. In this condition, Linalyl acetate was able to reverse the increase in blood pressure and reduce biomarkers associated with vascular damage, such as inflammation and oxidative stress [105].

In the context of diabetes mellitus, Linalyl acetate was evaluated in diabetic animals subjected to immobilization. This study used metformin as a positive control. Linalyl acetate treatment decreased plasma glucose concentration and improved ACh-induced vasorelaxation, similar to metformin. Interestingly, linalyl acetate increased the expression of eNOS and decreased the expression of NF-κB, improving endothelial function. These effects were superior to those of metformin treatment [106], corroborating the findings that show the vasculoprotective effects of this monoterpene [26,105]. These findings highlight its potential as an adjuvant therapy in vascular complications of different pathophysiological conditions involving the cardiovascular system.

### 4.6. Carvone

Carvone is a monoterpene ketone present in the essential oil of plants such as *Mentha* spp., *Origanum* spp., *Rosmarinus* spp., and *Thymus* spp. Highlighted carvone effects include neuroprotective [74], antidiabetic [107], antiproliferative [108,109], antioxidant, anti-inflammatory [110], antiarrhythmic [111], and vasorelaxant [32]. Recent research has elucidated its distinct mechanisms of vascular action on the nervous and cardiovascular systems, revealing therapeutic potential for regulating vascular function.

Ischemic stroke arises predominantly from occlusion of the middle cerebral artery, triggering inflammation, microvascular dysfunction, and altered blood–brain barrier permeability that contribute to neuronal injury [112]. Interestingly, carvone demonstrates significant efficacy in models of cerebral ischemia and reperfusion, acting through three distinct mechanisms. Initially, animals pretreated with carvone showed increased levels of antioxidant enzymes such as SOD, GSH, and CAT. Concurrently, this monoterpene reduced neuroinflammation by decreasing pro-inflammatory cytokines such as TNF-α, IL-1β, IL-6, and IL-10. Additionally, carvone modulates apoptotic pathways by suppressing TLR4 and caspase-1, providing comprehensive neuronal protection [74].

In rat thoracic arteries, carvone also exerts a beneficial effect by mediating vasorelaxant responses. The mechanism responsible for this effect involves blocking calcium influx through Cav1.2 [32]. Similar effects were observed in cardiomyocytes, where carvone was shown to reduce calcium influx by blocking Cav1.2, consequently reducing cardiac contractility [113]. The clinical implications of these findings are significant, pointing to possible future applications in the treatment of stroke, arterial hypertension, and various cardiac arrhythmia.

### 4.7. α-Terpineol

α-terpineol is an alcoholic monoterpene present in essential oils of plants such as *Ravensara aromatica*, *Melaleuca qinquenervia*, *Croton sonderianus* and *Eucalyptus* globulus [114,115]. It is described in the literature for a wide range of biological effects, such as anticancer [116], analgesic [117], antioxidant and anti-inflammatory [118], antihypertensive [29,60], and cardioprotective [119]. Pharmacological studies reveal that the beneficial effects of this monoterpene on vascular function are mediated by the interaction of vasoactive mechanisms.

Vascular tone plays an important role in regulating blood pressure. Maintaining vascular tone depends on the control of VSMC contraction and can be modulated by various substances [120]. In some pathophysiological conditions, vascular tone control may be compromised, directly interfering with blood pressure control. Therefore, substances that act through these mechanisms become important.

Studies on the vasorelaxant activity of α-terpineol revealed that this monoterpene modulates vascular tone through two distinct mechanisms. Initially, it was demonstrated that vasorelaxation, at least in part, is mediated by the vascular endothelium, with activation of the NO/sGC/PKG signaling pathway leading to relaxation of VSMCs [29,121]. Subsequently, Sabino and colleagues described that the vasorelaxant action of α-terpineol occurs as a result of inhibition of Ca^2+^ influx through Cav1.2 and ROCs [60]. This pharmacological duality reflects the complexity of its mechanisms of action, which involve both vascular endothelium-dependent and -independent pathways.

Important effects have also been described regarding the regulation of blood pressure by α-terpineol. In normotensive rats, α-terpineol dose-dependently reduced mean arterial pressure (MAP), accompanied by reflex tachycardia [29]. The blood pressure regulation effect was consistent in an arterial hypertension model. Treatment with α-terpineol in hypertensive animals induced by L-NAME administration was able to decrease MAP. In addition to its hemodynamic effects, α-terpineol has been shown to reverse the oxidative stress characteristic of hypertensive states. Treatment of hypertensive animals with this monoterpene significantly increased the levels of antioxidant factors such as CAT and GPx [60]. Therefore, this monoterpene may be a promising alternative for the treatment of hypertension.

### 4.8. Linalool

Linalool is an alcoholic monoterpene present in plants of the family Lamiaceae (genus *Lavandula*), *Lauraceae* (genus *Cinnamomum*), and Apiaceae (genus *Coriandrum*) [122]. Linalool presents numerous biological activities, such as anti-inflammatory [123], antiproliferative [124], and antioxidant [125]. In recent years, linalool has gained notoriety for its effects on vascular function and antihypertensive effects.

Similarly to other monoterpenes, linalool exerts multiple mechanisms on the regulation of vascular tone. The vasorelaxant effect of linalool was evaluated in the superior mesenteric artery, where it was shown to alter calcium mobilization in smooth muscle cells through Cav1.2 and IP3R, causing relaxation of VSMCs [61]. In the thoracic aorta, linalool-induced relaxation involves the release of NO through activation of the K^+^ channel [28]. The duality of the effect presented may be related to the different vascular beds studied, given that there is a difference in the expression of receptors and ion channels in the different vascular beds.

The effects of linalool on the vasculature are reflected in blood pressure regulation. Intravenous bolus administration of linalool has been shown to induce hypotension and reflex tachycardia. This effect may be attributed in part to the activation of muscarinic receptors [61].

In addition to its acute effects on vascular tone, linalool demonstrates antihypertensive and vascular remodeling properties. In the 2K1C hypertension model, treatment with linalool has been shown to reduce blood pressure through mechanisms related to increased NO bioavailability [61]. Furthermore, the beneficial effects on hypertension may be related to the downregulation of muscarinic receptor expression and blockade of the AMPK (ERK/JNK/p38) cascade, a signaling pathway involved in VSMC hypertrophy [5].

It is important to highlight that various delivery systems are used to increase the specificity, potency, and reduce the toxicity of monoterpenes. Therefore, the antihypertensive activity of linalool was evaluated after complexing this monoterpene with β-cyclodextrin. In SHR, a 20-day treatment with linalool incorporated with β-cyclodextrin potentiated the antihypertensive effect and reduced it. Furthermore, it was observed that this preparation was able to reduce the Phe-induced increase in contractility and improve the NO pathway response in VSMC [75]. Therefore, linalool has excellent effects on the reparative mechanisms associated with hypertension-related vascular remodeling [8].

### 4.9. Perillyl Alcohol

First extracted from herbs of the genus Perilla, perillyl alcohol is a monoterpene found in the essential oils of various plants such as peppermint, spearmint, lavender, bergamot, lemongrass, thyme, and rosemary [126]. Reports describe perillyl alcohol as having anticancer [127], anti-inflammatory [128], antioxidant [129,130], and cardioprotective [63,131] properties. Recent studies have elucidated promising mechanisms on the vasculature, which include vasorelaxant, antioxidant, and anti-inflammatory actions.

Research demonstrates that perillyl alcohol exerts significant vasodilatory effects through mechanisms predominantly independent of the endothelium. In rat aortic arteries, perillyl alcohol showed greater pharmacological potency under conditions of depolarization induced by KCl or BayK-8644, suggesting a possible direct action on Ca_v_ [63]. This mechanism was confirmed in porcine coronary arteries, where perillyl alcohol inhibited contractions induced by U-46619, 5-HT, or KCl [132]. Thus, the blockade of the Cav seems essential for this monoterpene’s vasorelaxant effect.

Perillyl alcohol, similar to other monoterpenes, also exerts neuroprotective effects. Using a model of ischemia and reperfusion in the middle cerebral artery, pretreatment with perillyl alcohol reduced neurological damage and infarct volume. These benefits are associated with decreased lipid peroxidation and increased activity of SOD, GSH, and GPx, decreased levels of pro-inflammatory cytokines such as IL-1β, TNF-α, and IL-6, and reduced expression of COX-2, iNOS, and NF-κB. This demonstrates the crucial role of antioxidant and anti-inflammatory effects in neuroprotection induced by perillyl alcohol [128].

Furthermore, perillyl alcohol modulates pulmonary hypertension (PAH). Studies in models of monocrotaline-induced pulmonary hypertension have demonstrated that three-week treatment reverses pulmonary artery vascular remodeling by inhibiting vascular cell apoptosis, restoring the Bax/Bcl-2 ratio, reducing oxidative stress by increasing antioxidant enzymes such as SOD, CAT, and GPx, and reducing inflammation by decreasing the pro-inflammatory cytokines TNF-α and IL-6 [131].

### 4.10. Borneol

Borneol is a monoterpenoid alcohol found in plants like *Valeriana officianalis*, *Matriaca chamomilla*, and *Lavandula officinalis* [3]. Several reports indicate borneol is a promising candidate for various brain diseases due to its ability to cross the blood–brain barrier [133,134,135]. Moreover, borneol also has anti-inflammatory [136], vasorelaxant [23], antioxidant, antihypertensive [137], and antidiabetic [138] effects.

In the vasculature, borneol induces a relaxant effect in the thoracic aorta of rats in an endothelium-independent manner, modulating intracellular calcium concentration. The mechanism underlying borneol’s vasorelaxation likely involves Cav1.2 blockade, intracellular calcium mobilization, and activation of potassium channels [67]. Furthermore, borneol induced relaxation with the participation of Kir3 channels in VSMCs [23], similar to that described by Silva-Filho et al. [67].

In contrast, Santos et al. demonstrated an endothelium-dependent vasorelaxant effect in the aorta of rats. The mechanism involves the production of NO and prostanoids, as demonstrated by experiments in the presence of L-NAME and indomethacin [23]. This dual effect demonstrated by borneol has also been reported for other monoterpenes. It has been associated with beneficial effects, given that this monoterpene is still capable of exerting its effects in cases of endothelial dysfunction.

Neuroprotective effects have been associated with borneol. Interestingly, in a model of cerebral ischemia, borneol increased the expression of HIF-α and VEGF, resulting in angiogenesis and protection of the blood–brain barrier. Therefore, these effects restore neuronal blood flow and lead to neuroprotection [134]. Therefore, the preliminary results obtained for borneol indicate that it is a promising molecule for further studies in models of endothelial dysfunction.

## 5. Translational Challenges: Bioavailability, Clinical Evidence, and Safety

Although monoterpenes demonstrate promising vascular effects in preclinical models, several translation barriers must be addressed before clinical application. One of the main concerns is bioavailability. Monoterpenes are often volatile and lipophilic, which favors transdermal and inhalation absorption but limits their oral absorption [139]. This is the most compromised pharmacokinetic aspect. Furthermore, they undergo extensive metabolism in the liver, potentially producing inactive or unknown metabolites [140]. Furthermore, they have rapid clearance and a short elimination half-life, precluding the accumulation of their metabolites [139,140]. However, studies describe the pharmacokinetics of some monoterpenes; more robust trials are needed, especially regarding long-term treatment [141,142].

To overcome challenges related to bioavailability, nanoformulations have been developed in recent years. Some studies have shown that the inclusion of β-cyclodextrin with monoterpenes can produce significant improvements in chemical and pharmacological properties. In particular, this approach significantly improves solubility, making the compounds more suitable for oral administration. These systems have demonstrated increased potency for monoterpenes, such as linalool [75] and β-pinene [66]. Furthermore, chemical modifications of monoterpene molecules have been suggested to improve pharmacokinetic and pharmacological properties [143].

Another critical gap is the limited clinical evidence. Although monoterpenes like menthol, limonene, and carvacrol have been widely used in traditional medicine and food applications [4], controlled clinical trials assessing their efficacy on vascular function are virtually nonexistent. This limits the ability to extrapolate preclinical findings to human health. Establishing robust clinical data through dose-escalation and efficacy trials will be essential for their future therapeutic validation.

Safety is a further concern, especially at high concentrations required for therapeutic effect. While many monoterpenes are classified as Generally Recognized as Safe (GRAS) for food use, chronic exposure, high-dose supplementation, or intravenous use may pose toxicity risks. Potential adverse effects include gastrointestinal discomfort, hepatotoxicity, or neurotoxicity, depending on the compound and route of administration [144]. Therefore, comprehensive toxicological studies are needed to assess long-term safety and define acceptable daily intake limits [145].

Overcoming these challenges requires the development of optimized delivery systems, such as nanoformulations or prodrugs, to enhance stability, target specificity, and therapeutic window [146]. Addressing these translational aspects is crucial to transitioning monoterpenes from promising phytochemicals into validated agents for vascular protection.

## 6. Study Limitations, Inconsistencies, and Gaps in Knowledge

Monoterpenes exhibit broad vascular activity. However, some limitations need to be addressed to generate more robust data for clinical application. Most of the studies presented here rely heavily on rodent models. While these offer valuable mechanistic insights, significant physiological differences between rodents and humans pose challenges for clinical translation.

Such differences include vascular architecture: mice have a relatively thin intimal layer, while humans have more complex layers [147]. Differences in receptor and ion channel expression also exist across vascular beds in rodents versus humans [148]. For example, human VSMCs regulate contractility through voltage-sensitive calcium channels of the Cav1.2, Cav3.2, and Cav3.3 types. In rodents, VSMC contractility is mediated by currents induced by Cav1.2 and Cav3.2 channels, which generate larger calcium inward currents compared to humans [149]. Additionally, vascular lesions can develop in different locations between species. For example, atherosclerotic lesions in rats occur predominantly in the aortic sinus, with relative protection of the coronary arteries, whereas humans develop lesions more frequently in the coronary arteries, carotid arteries, and peripheral vessels [147].

Furthermore, many vascular effects have been demonstrated at very low concentrations, raising concerns about the feasibility of achieving sufficient plasma levels to achieve pharmacological effects in humans through conventional administration routes due to bioavailability issues. Pharmacokinetic and toxicological data are thus urgently needed to determine therapeutic windows and systemic bioavailability.

Second, discrepancies in the literature regarding the mechanisms of action of specific monoterpenes remain unresolved. Some compounds, such as linalool or carvacrol, exhibit endothelium-dependent relaxation in certain vascular beds while showing endothelium-independent effects in others [28,29,40,43]. These inconsistencies may stem from variations in vascular bed physiology, species-specific responses, and divergent experimental methodologies, including differences in precontracting agents, inhibitors used, and ex vivo tissue conditions [34,37]. Future studies should prioritize standardized protocols to clarify these discrepancies.

Finally, notable gaps in knowledge persist. The metabolism of monoterpenes remains poorly understood; most studies focus exclusively on the parent compounds, ignoring the potential role of active metabolites generated in vivo [150]. Additionally, while select monoterpenes have been extensively characterized, others—such as rotundifolone [44], perillyl alcohol [63,151], carvone [152], and sabinene [153]—are relatively understudied despite preliminary evidence of vascular activity. Additionally, certain monoterpenes, like verbenone and α-fenchene, have not yet been examined in relation to the vasculature, even though they demonstrate significant antioxidant and anti-inflammatory potential [154,155,156,157]. Moreover, several proposed mechanisms, including TRP channel modulation and EDHF-related pathways, lack a detailed understanding of the molecular targets involved [35,36,42]. Future investigations should incorporate metabolomic approaches, high-throughput screening for target identification, and expanded evaluation of lesser-known monoterpenes in vascular models.

Finally, despite their therapeutic potential, the long-term safety of monoterpenes remains unclear, with few studies evaluating cumulative toxicity or target organ effects after months of use. Furthermore, the lack of standardization in test concentrations, animal models, and administration routes compromises the robustness and reproducibility of results.

These considerations highlight the importance of ongoing research to bridge preclinical findings and translational potential, ultimately guiding the development of safe and effective monoterpene-based therapies for vascular diseases.

## 7. Conclusions

Terpenes correspond to 90% of essential oils, the largest group of secondary metabolites in aromatic plants. Monoterpenes, a selected group of terpenes, effectively control vascular tone, reduce oxidative stress, attenuate inflammation, vascular remodeling, and prevent pathophysiological conditions. Moreover, monoterpenes attracted the pharmaceutical industry’s interest due to their low synthesis costs. Therefore, understanding monoterpenes as promising molecules can lead to the development of new pharmacological tools to control the main vascular biological events in health and disease.

In this review, we observed that monoterpenes citral, linalyl acetate, α-terpineol, eugenol, and borneol exert a biological effect on the vascular endothelium. The participation of the NO pathway is constant in the impact of monoterpenes that act on the vascular endothelium. On the other hand, the monoterpenes linalool, geraniol, citronellal, citronellol, α-terpineol, carvacrol, thymol, carveol, carvone, eugenol, menthol, terpinen-4-ol, limonene, rotundifolone, perillyl alcohol, α-pinene, and borneol exert an action on the vascular musculature, mainly with the participation of calcium channels (Cav) and TRPs. In this context, vasodilation has been achieved with significant efficiency without the specificity of the vascular bed. Regarding in vivo experiments, the most promising results were obtained with terpineol, carvacrol, linalyl acetate, and linalool, demonstrating a bradycardic and BP-lowering action. Another point that has been extensively investigated over the years has been antioxidant action. In this sense, it has been observed that most monoterpenes maintain this activity.

Given the evidence presented, monoterpenes emerge as promising molecules with beneficial potential in modulating endothelial function. The diversity of molecular mechanisms involved—including activation of the nitric oxide pathway, modulation of ion channels, and antioxidant action—reinforces their pharmacological value. However, although preclinical studies indicate promising effects, there are still important gaps to be filled. Therefore, future studies should focus on the detailed elucidation of the specific molecular targets of monoterpenes that do not yet present their detailed mechanisms. In addition, the development of formulations that optimize their therapeutic efficacy and robust clinical trials that validate their translational application will be of great scientific value. Progress in this direction may consolidate monoterpenes as pharmacological agents for the treatment of conditions related to vascular dysfunction associated with oxidative stress and inflammation.

## Figures and Tables

**Table 1:** Description of the main characteristics of preclinical studies using monoterpenes with action on vascular function.

		Estudos In Vitro		
Monoterpene	Experimental Model	Concentration Tested	Mechanism of Action	**Reference**
Geraniol	Thoracic aorta of diabetic rats	10–300 µmol/L	Vasorelaxation by inhibiting Ca_v_1.2 and ROC	[51]
	HUVEC	25–100 µmol/L	Inhibits Ox-LDL-induced inflammation and oxidative stress by targeting PI3/AKT/NRF2	[52]
Carvacrol	Rat thoracic aorta artery	1–1 × 10^3^ µmol/L	Vasorelaxation by block the Ca^2+^ influx through the membrane	[53]
	Posterior cerebral or cerebellar arteries from rats	10–1 × 10^4^ µmol/L	Relaxation induced by Ca^2+^ influx via TRPV3 channels in the endothelium and activation of K_ca_2.3 and K_ca_3.1 channels	[40]
	Rat thoracic aorta artery	0.01–100 µmol/L	Attenuation of the vasoconstrictor action via ROS inhibition and NOS stimulation	[31]
	Superior mesenteric artery of rats	0.01–300 µmol/L	Vasorelaxation by inhibition of the Ca^2+^ influx through Ca_v_1.2, ROC and SOC channels.	[43]
Citronellal	Superior mesenteric artery of rats	1–1 × 10^5^ µmol/L	Vasorelaxation by inhibiting calcium influx	[54]
Citronellol	Superior mesenteric artery of rats	640–1.9 × 10^6^ µmol/L	Vasorelaxation by inhibiting calcium influx and Ca_v_1.2	[55]
Linalyl acetate	Rabbit carotid artery	300 µmol/L	Vasorelaxation by stimulating eNOS in vascular endothelium and stimulating MLCP in vascular smooth muscle	[56]
	HUVEC	509 µmol/L	Blocked the Ca^2+^ influx in endothelial cells	[57]
Citral	Thoracic aorta of rats from hypertensive rats	6.24–6.24 × 10^3^ µmol/L	Vasorelaxation by NO/cyclic GMP pathway and inhibiting Ca_v_1.2	[58]
	Rat thoracic aorta	300–3 × 10^4^ µmol/L	Vasorelaxation by NO/cyclic GMP pathway and the calcium influx through Ca_v_1.2	[59]
	Rat thoracic aorta	6.57–6.57 × 10^3^ µmol/L	Vasorelaxation by NO/cyclic GMP pathway and inhibiting Ca_v_1.2	[24]
Carvone	Rat thoracic aorta	100 µmol/L	Vasorelaxation by blocking Ca_v_1.2	[32]
α-terpineol	Superior mesenteric artery of Wistar rats	1 × 10^−6^–10 µmol/L	Vasorelaxation by NO/cyclic GMP pathway	[29]
	Superior mesenteric artery of hypertensive rats	1.10^−4^–1 × 10^4^ µmol/L	Vasorelaxation by inhibiting Ca_v_1.2	[60]
Linalool	Rat superior mesenteric artery	6.4–6.4 × 10^3^ µmol/L	Vasorelaxation by inhibiting calcium influx and Ca_v_1.2	[61]
	Rat thoracic aorta	100 µmol/L	Vasorelaxation by blocking Ca_v_1.2 and elevating NO	[32]
	Mouse thoracic aortas	10–500 µmol/L	Vasorelaxation by activating sCG and K^+^ channels.	[28]
	Ang II-induced VSMCs	50–150 µmol/L	Inhibited the proliferation and migration by inhibithing MAPK	[5]
p-cymene	Rat thoracic aorta artery	1–1 × 10^3^ µmol/L	Vasorelaxation by activation Kir2 and Kir6 Channels	[62]
Thymol	Rat thoracic aorta artery	1–1 × 10^3^ µmol/L	Vasorelaxation by block the Ca^2+^ influx through the membrane	[53]
Carveol	Rat thoracic aorta artery	1–5 × 10^3^ µmol/L	Vasorelaxation by inhibiting Ca_v_1.2 channels	[63]
	Human umbilical artery	1–5 × 10^3^ µmol/L	Vasorelaxation by inhibiting Ca_v_1.2 and partial participation of K_ca_1.1 channels	[64]
Perillyl alcohol	Rat thoracic aorta artery	1–5 × 10^3^ µmol/L	Induced relaxant effect by inhibition of PKC and IP_3_ pathway	[63]
	Human vascular smooth muscle cells	100–2 × 10^3^ µmol/L	Inhibits proliferation and also induces apoptosis	[65]
β-pinene	Superior mesenteric artery of rats	0.1–3 × 10^4^ µmol/L	Vasorelaxant effect involve blocking Ca^2+^ influx through the Ca_v_1.2 channels, associated with decreased sensitivity of contractile machinery to Ca^2+^	[66]
Borneol	Rat thoracic aorta artery	1 × 10^−3^–300 µmol/L	Vasorelaxation by calcium influx blockade through Ca_v_1.2 channels, calcium mobilization from intracellular stores and potassium channels activation.	[67]
	Rat thoracic aorta artery	1 × 10^−4^–300 µmol/L	Vasorelaxant effect with the participation of NO and prostanoids in vascular endothelium and action on the VSMC dependent in Kir6 channels.	[23]
**Estudos In Vivo**				
**Monoterpene**	**Experimental Model**	**Dose Tested**	**Mechanism of Action**	**Reference**
Geraniol	Mice fed with a high-fat diet	100 mg/kg/day (intraperitoneally)	Improves endothelial function by inhibiting NOX-2 derived ROS generation	[68]
Carvacrol	Normotensive rats	1–20 mg/kg (intravenous)	Induced hypotension, bradycardia, and negative inotropic and chronotropic effects	[43]
	Diabetic rats	10–20 mg/kg/day (intraperitoneally)	Reduced hypercontractility by activating the PI3K/Akt signaling pathway	[69]
	Spontaneously hypertensive rats (SHR)	50–100 mg/kg/day (oral)	Improved reendothelialization by increasing eNOS expression and reducing senescence and oxidative stress in endothelial progenitor cells.	[70]
Citronellal	Rats fed with a high-fat diet	50–150 mg/kg/day	Improved endothelial dysfunction, increased cell migration, and suppressed oxidative stress and inflammation in vascular endothelium	[71]
	Diabetic rats	150 mg/kg/day	Increased expression of S1P1 and eNOS, accompanied by increased SOD levels and ROS reduction.	[72]
	Diabetic rats	50–150 mg/kg/day	Suppressed the expression of NHE1 and TPRM2, alleviated oxidative stress-induced mitochondrial damage	[73]
Linalyl acetate	Hypertension induced by immobilization stress and intraperitoneal injection of nicotine	25–100 mg/kg	Suppression of phosphorylation and activation of the NADPH oxidase, decrease in ROS production and increased expression of eNOS	[26]
Carvone	Cerebral I/R injury in rats	1–20 mg/kg/day (intraperitoneally)	It had antioxidative, anti-inflammatory, and anti-apoptotic effects against cerebral I/R brain injury.	[74]
α-terpineol	Normotensive rats	1–30 mg/kg (oral)	Dose-dependent hypotension followed by reflex tachycardia	[29]
	Hypertension induced by L-NAME	25–100 mg/kg/day (intraperitoneally)	Reduce arterial pressure, decrease vascular resistance, and restore enzymatic antioxidants	[60]
Linalool	Normotensive rats	1–20 mg/kg/day (intravenous)	Hypotension and bradycardia attenuated by inhibition of muscarinic receptors	[61]
	Hypertensive rats (two kidneys and a clip–2R1C)	200 mg/kg/day (oral)	Reduced blood pressure without changing the heart rate	[61]
	SHR	50–100 mg/kg/day (oral)	Reduced blood pressure, increased levels of the anti-inflammatory cytokine (IL-10) and improved vasodilator responsiveness	[75]
α-Pinene	Aorta artery from ApoE/mice	Particulate matter in ratios of 10:1:1	Increased vascular expression of HO-1, MMP-9 and ET-1	[76]
β-Pinene	Hypertension induced by L-NAME	200 mg/kg	Reduce arterial pressure	[66]

Voltage-gated L-type calcium channels (Cav); Nitric oxide (NO); Soluble guanylyl cyclase (sCG); Mitogen-activated protein kinase (MAPK); Receptor-operated calcium channels (ROC); Sphingosine-1-phosphate receptor subtype 1 (S1P1); Endothelial nitric oxide synthase (eNOS); (SOD); Reactive oxygen species (ROS); Na^+^/H^+^ exchanger (NHE1); Transient receptor potential melastatin channel (TPRM); Guanosine monophosphate (GMP); Myosin light chain phosphatase (MLCP); Nicotinamide adenine dinucleotide phosphate (NADPH); Transient receptor potential vanilloid (TRPV); Calcium-activated potassium channels (Kca); Superoxide dismutase (SOC); Protein kinase B (PI3K or Akt); ATP-sensitive potassium channels (Kir); Cyclic Adenosine Monophosphate (cAMP); Cyclic guanosine monophosphate (cGMP); Phosphodiesterase (PDE); Protein kinase C (PKC); Inositol 1,4,5-trisphosphate (IP_3_); Heme oxygenase-1 (HO-1); Matrix metallopeptidase (MMP); endothelin-1 (ET-1); Angiotensin converting enzyme (ACE); Vascular smooth muscle cells (VSMC).

## Data Availability

No new data were created or analyzed in this study. Data sharing is not applicable to this article.

## Abbreviations

The following abbreviations are used in this manuscript:

ACE	Angiotensin converting enzyme
Ach	Acetylcholine
AMPK	AMP-activated protein kinase
BH_4_	Tetrahydrobiopterin
cAMP	Cyclic Adenosine Monophosphate
CAT	Catalase
Ca_v_	Voltage-gated calcium channels
cGMP	Cyclic guanosine monophosphate
COX	Cyclooxygenase
DMAPP	Dimethylallyl diphosphate
DRP1	Dynamin-related protein 1
DXP	1-Deoxy-d-xylulose5-phosphate
EDHF	Endothelium-derived hyperpolarizing factors
eNOS	Endothelial nitric oxide synthase
EPC	Endothelial progenitor cell
ET-1	Endothelin-1
G3P	Glyceraldehyde-3-phosphate
GMP	Guanosine monophosphate
GPP	Geranyl diphosphate enzyme
GPx	Glutathione peroxidases
GSH	Glutathione reduced
GTP	Guanosine triphosphate
HIF-α	Hypoxia-inducible factor 1-alpha
HO-1	Heme oxygenase-1
ICAM-1	Intercellular Adhesion Molecule 1
IL	Interleukins
INDO	Indomethacin
iNOS	Induced nitric oxide synthase
IP_3_	Inositol 1,4,5-trisphosphate
IP_3_R	Inositol 1,4,5-trisphosphate receptor
IPP	Isopentenyl diphosphate
K_Ca_	Calcium-activated potassium channels
K_Ca_2.3-SK_Ca_	Potassium channels activated by small calcium conductance
K_Ca_3.1-IK_Ca_	Potassium channels activated by intermediate calcium conductance
Kir	ATP-sensitive potassium channels
K_v_	Voltage-operated K^+^ channels
LDL	Low-density lipoprotein
L-Name	Nitro-L-arginine methyl ester (NOS inhibitors)
MAP	Mean arterial pressure
MAPK	Mitogen-activated protein kinase
MCT	Monocrotaline
MEP	4-phosphate methylerythritol
MLCP	Myosin light chain phosphatase
MMP	Matrix metallopeptidase
MVA	Mevalonate
MW	Molecular Weigh
NADPH	Nicotinamide adenine dinucleotide phosphate
NF-κB	Factor nuclear kappa B
NHE1	Na^+^/H^+^ exchanger
NO	Nitric oxide
NQO1	Quinone oxidoreductase-1
Nrf-2	Nuclear factor 2 related to erythroid factor 2
PAH	Pulmonary hypertension
PDE	Phosphodiesterase
PI_3_K	Phosphatidylinositol 3-kinase
PKC	Protein kinase C
PKG	Cyclic GMP-dependent protein kinase
ROC	Receptor-operated calcium channels
ROS	Reactive oxygen species
RyR	Ryanodine receptor
S1P1	Sphingosine-1-phosphate receptor subtype 1
sCG	Soluble guanylyl cyclase
SCI	Spinal cord injury
SERCA	Sarcoendoplasmic reticulum calcium ATPase
SMC	Smooth muscle cells
SOC	Store-operated channels
SOCE	Store-operated calcium entry
SOD	Superoxide dismutase
SR	Sarcoplasmic reticulum
STZ	Streptozotocin
TEA	Tetraethylammonium chloride
TLR4	Toll-like receptor 4
TNF-α	Protein tumor necrosis α
TPRM	Transient receptor potential melastatin channel
TPVR	Total peripheric vascular resistance
TRP	Transient receptor potential
TRPV	Transient receptor potential vanilloid
VCAM-1	Vascular Cell Adhesion Molecule 1
VEGF	Vascular endothelial growth fator
VSMC	Vascular smooth muscle cells
